# 

**Published:** 1892-06

**Authors:** 


SUBSCRIBE FOR THE

### International Dental Journal,

THE ORGAN OF THE PROFESSION.

### Published by the International Dental Publication Company.

LOUIS JACK, President, Philadelphia.
BENJAMIN LORD, Vice-President, New York.
C. N. PEIRCE, Treasurer, Philadelphia.
GEO. S. ALLEN, Secretary, 51 West Thirty-seventh St., New York.

**Publication Office, 716 Filbert St., Philadelphia, Pa.**

STOCKHOLDERS.

| | |
|---|---|
| Frank Abbott | New York, N.Y. |
| Geo. S. Allen | New York, N.Y. |
| W. W. Allport | Chicago, Ill. |
| R. R. Andrews | Cambridge, Mass. |
| H. A. Baker | Boston, Mass. |
| A. E. Baldwin | Chicago, Ill. |
| W. C. Barrett | Buffalo, N.Y. |
| A. P. Beale | Philadelphia, Pa. |
| S. T. Beale, Jr. | Philadelphia, Pa. |
| C. S. Beck | Wilkesbarre, Pa. |
| G. V. Black | Jacksonville, Ill. |
| C. F. W. Bodecker | New York, N.Y. |
| E. A. Bogue | New York, N.Y. |
| W. G. A. Bonwill | Philadelphia, Pa. |
| C. A. Brackett | Newport, R.I. |
| Thomas Bradley | New York, N.Y. |
| Edward C. Briggs | Boston, Mass. |
| Albert H. Brockway | Brooklyn, N.Y. |
| A. E. Brown | Chicago, Ill. |
| Wm. Carr | New York, N.Y. |
| Geo. H. Chance | Portland, Oregon. |
| G. F. Cheney | St. Johnsbury, Vt. |
| Dwight M. Clapp | Boston, Mass. |
| John T. Codman | Boston, Mass. |
| Chas. D. Cook | Brooklyn, N.Y. |
| J. N. Crouse | Chicago, Ill. |
| Edw. T. Darby | Philadelphia, Pa. |
| H. S. Draper | Boston, Mass. |
| Wm. H. Dwinelle | New York, N.Y. |
| A. R. Eaton | Elizabeth, N.J. |
| Chas. J. Essig | Philadelphia, Pa. |
| J. N. Farrer | New York, N.Y. |
| T. Fillebrown | Portland, Me. |
| W. B. Finney | Baltimore, Md. |
| T. A. Fletcher | New York, N.Y. |
| M. W. Foster | Baltimore, Md. |
| Chas. E. Francis | New York, N.Y. |
| E. C. Fundenberg | Pittsburg, Pa. |
| W. F. Fundenberg | Pittsburg, Pa. |
| W. H. Fundenberg | Pittsburg, Pa. |
| E. S. Gaylord | New Haven, Conn. |
| J. P. Geran | Brooklyn, N.Y. |
| A. H. Gilson | Boston, Mass. |
| S. H. Guilford | Philadelphia, Pa. |
| John I. Hart | New York, N.Y. |
| O. E. Hill | Brooklyn, N.Y. |
| J. F. P. Hodson | New York, N.Y. |
| J. Morgan Howe | New York, N.Y. |
| Robert Huey | Philadelphia, Pa. |
| A. O. Hunt | Iowa City, Iowa. |
| Louis Jack | Philadelphia, Pa. |
| V. H. Jackson | New York, N.Y. |
| C. R. Jefferis | Wilmington, Del. |
| C. A. Kingsbury | Philadelphia, Pa. |
| Edw. C. Kirk | Philadelphia, Pa. |
| C. R. E. Koch | Chicago, Ill. |
| W. T. La Roche | Harrington Park, N.J. |
| W. K. Leech | Philadelphia, Pa. |
| F. A. Levy | Orange, N.J. |
| Wilbur F. Litch | Philadelphia, Pa. |
| J. Bond Littig | New York, N.Y. |
| Benjamin Lord | New York, N.Y. |
| Geo. W. Lovejoy | Montreal, Ont. |
| John S. Marshall | Chicago, Ill. |
| H. J. McKellops | St. Louis, Mo. |
| Charles McManus | Hartford, Conn. |
| James McManus | Hartford, Conn. |
| Dan'l Neal McQuillan | Philadelphia, Pa. |
| Horatio C. Meriam | Salem, Mass. |
| W. D. Miller | Berlin, Germany. |
| W. E. Page | Boston, Mass. |
| S. B. Palmer | Syracuse, N.Y. |
| C. B. Parker | Brooklyn, N.Y. |
| D. D. Peabody | Stoneham, Mass. |
| C. N. Peirce | Philadelphia, Pa. |
| S. G. Perry | New York, N.Y. |
| W. A. Phreaner | Philadelphia, Pa. |
| Chas. E. Pike | Philadelphia, Pa. |
| W. E. Pinkham | New York, N.Y. |
| W. H. Potter | Boston, Mass. |
| Chas. P. Pruyn | Chicago, Ill. |
| H. C. Register | Philadelphia, Pa. |
| Z. T. Sailer | New York, N.Y. |
| L. D. Shepard | Boston, Mass. |
| Geo. R. Shidle | Pittsburg, Pa. |
| Eugene H. Smith | Boston, Mass. |
| B. Holly Smith | Baltimore, Md. |
| H. A. Smith | Cincinnati, Ohio. |
| S. G. Stevens | Boston, Mass. |
| W. X. Sudduth | Minneapolis, Minn. |
| Eugene S. Talbott | Chicago, Ill. |
| Ambler Tees | Philadelphia, Pa. |
| J. D. Thomas | Philadelphia, Pa. |
| James Truman | Philadelphia, Pa. |
| Wm. H. Trueman | Philadelphia, Pa. |
| W. W. Walker | New York, N.Y. |
| I. F. Wardwell | Stanford, Conn. |
| G. W. Warren | Philadelphia, Pa. |
| T. S. Waters | Baltimore, Md. |
| Geo. W. Weld | New York, N.Y. |
| Julius G. W. Werner | Boston, Mass. |
| Jacob L. Williams | Boston, Mass. |
| R. B. Winder | Baltimore, Md. |
| C. A. Woodward | New York, N.Y. |
| J. A. Woodward | Philadelphia, Pa. |
| W. A. Woodward | New York, N.Y. |
| W. J. Younger | San Francisco, Cal. |

The officers of the International Dental Publication Company serve without salary, the investments of the stockholders merely guarantee the success of the enterprise, ALL PROFITS being devoted to the enlargement or improvement of the International Dental Journal.

The support of every dentist having the welfare of his profession at heart is earnestly solicited. Subscription, $2.50 per Annum.

*THE INTERNATIONAL DENTAL PUBLICATION CO.*

